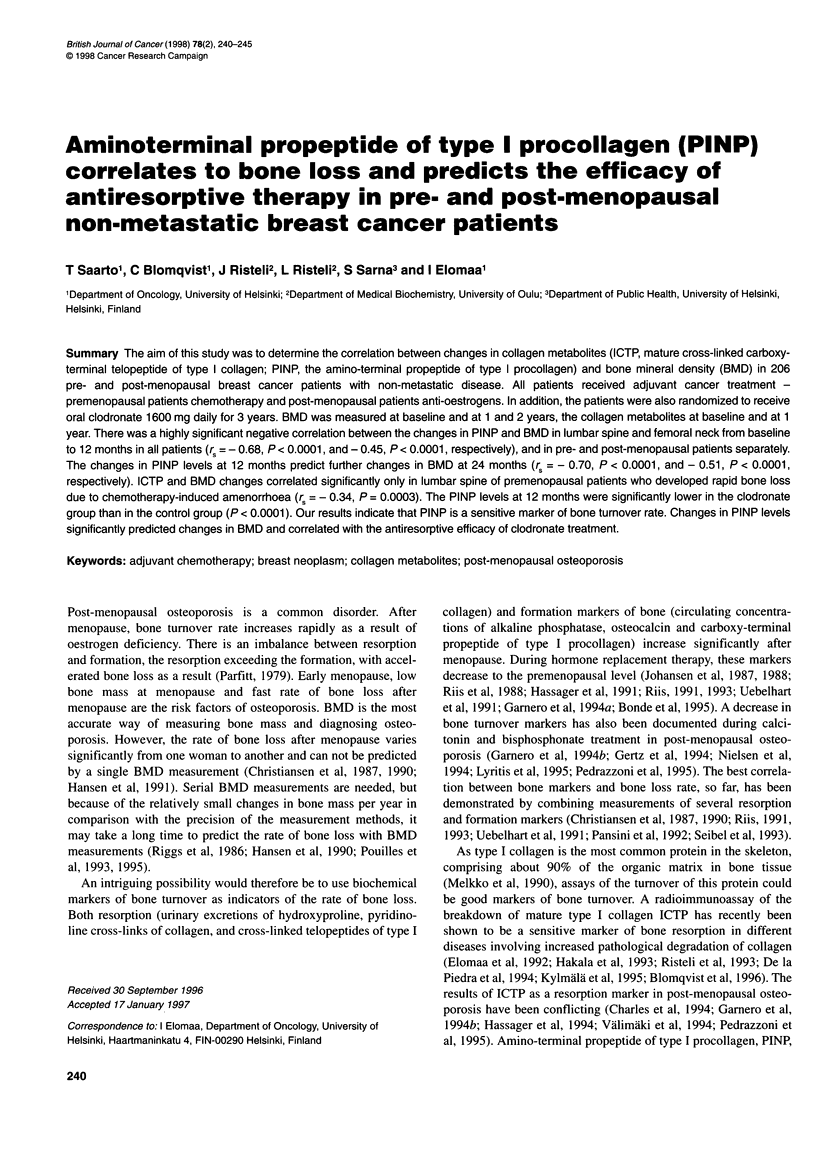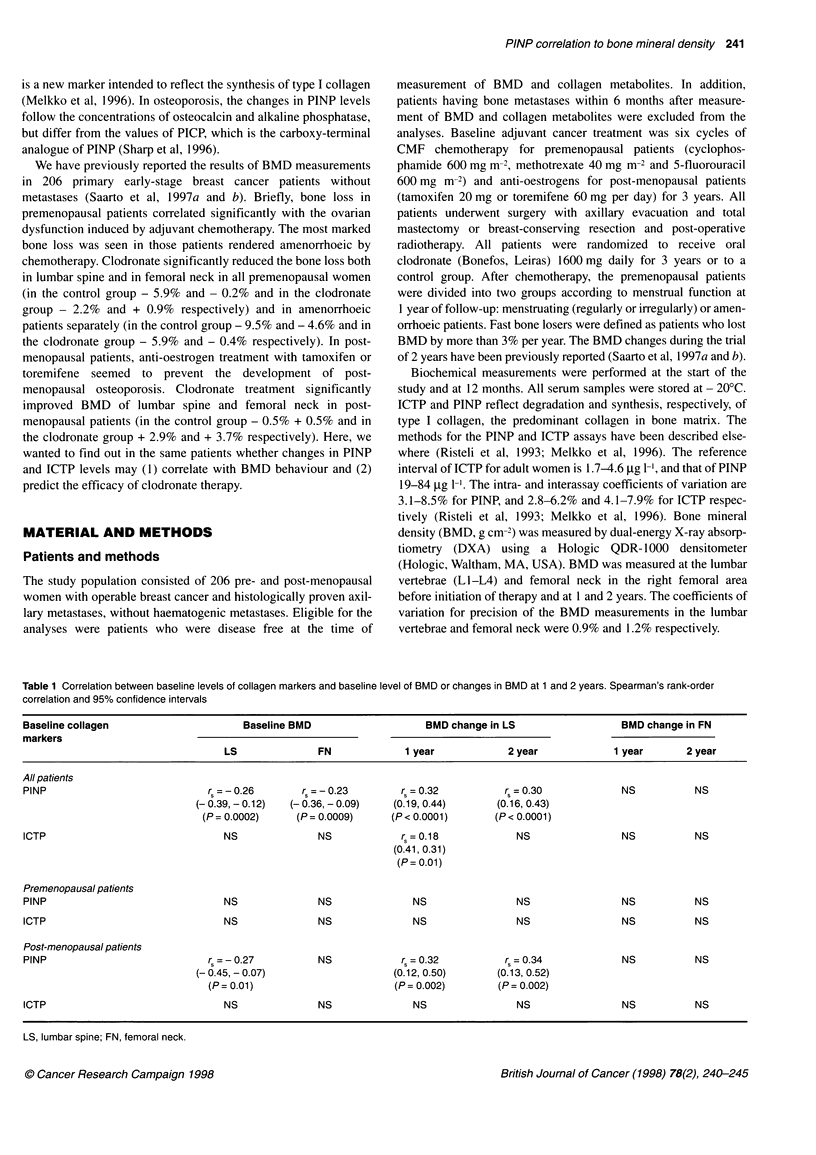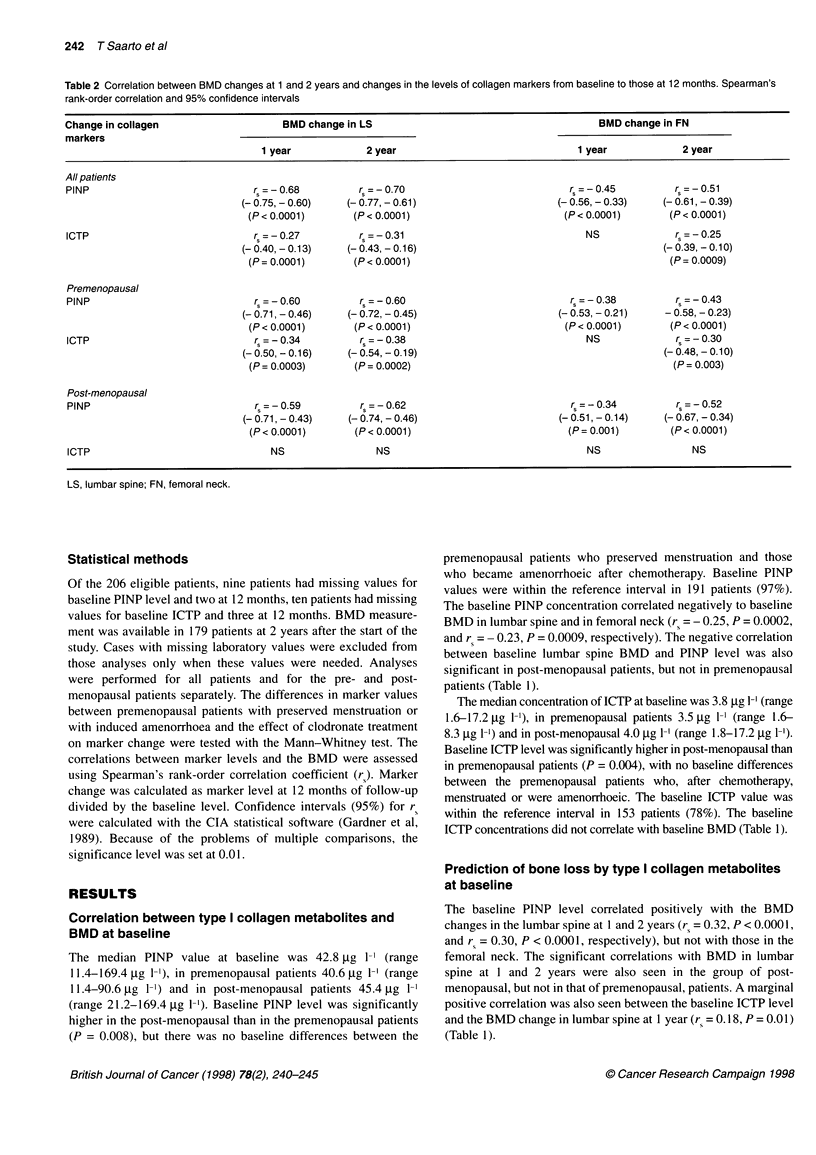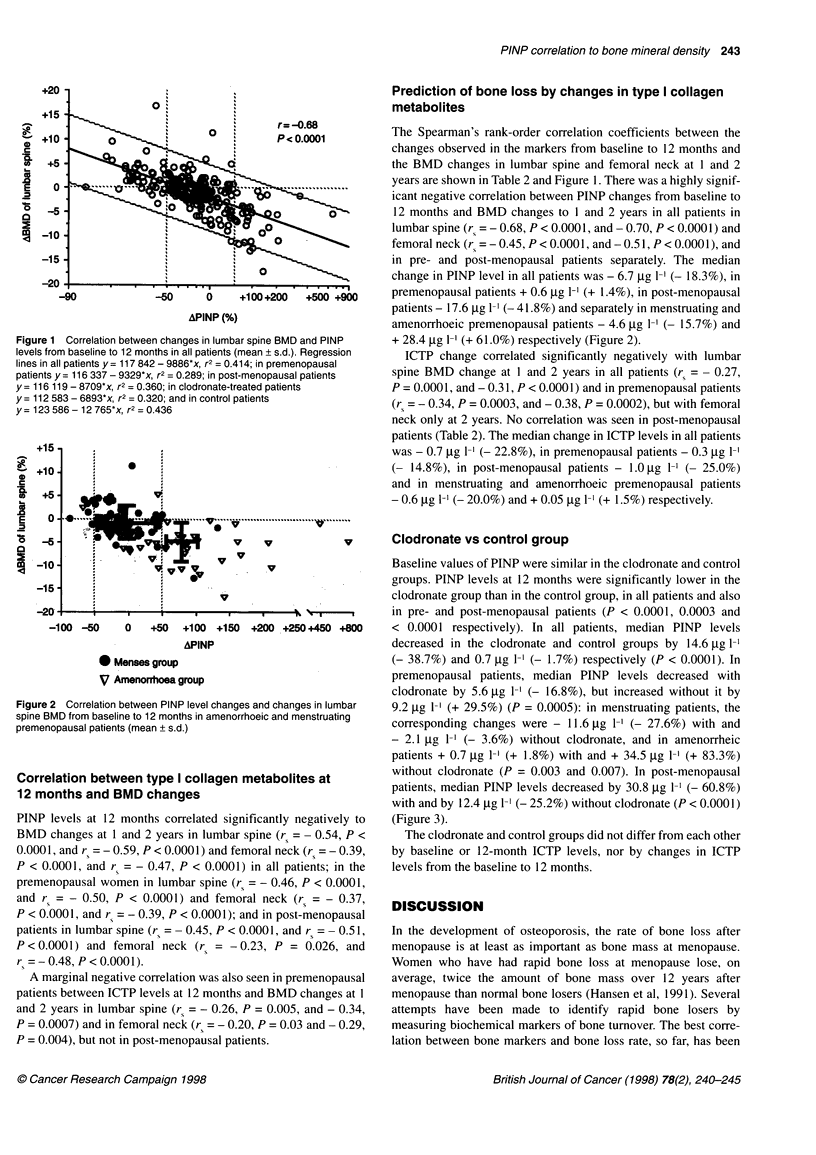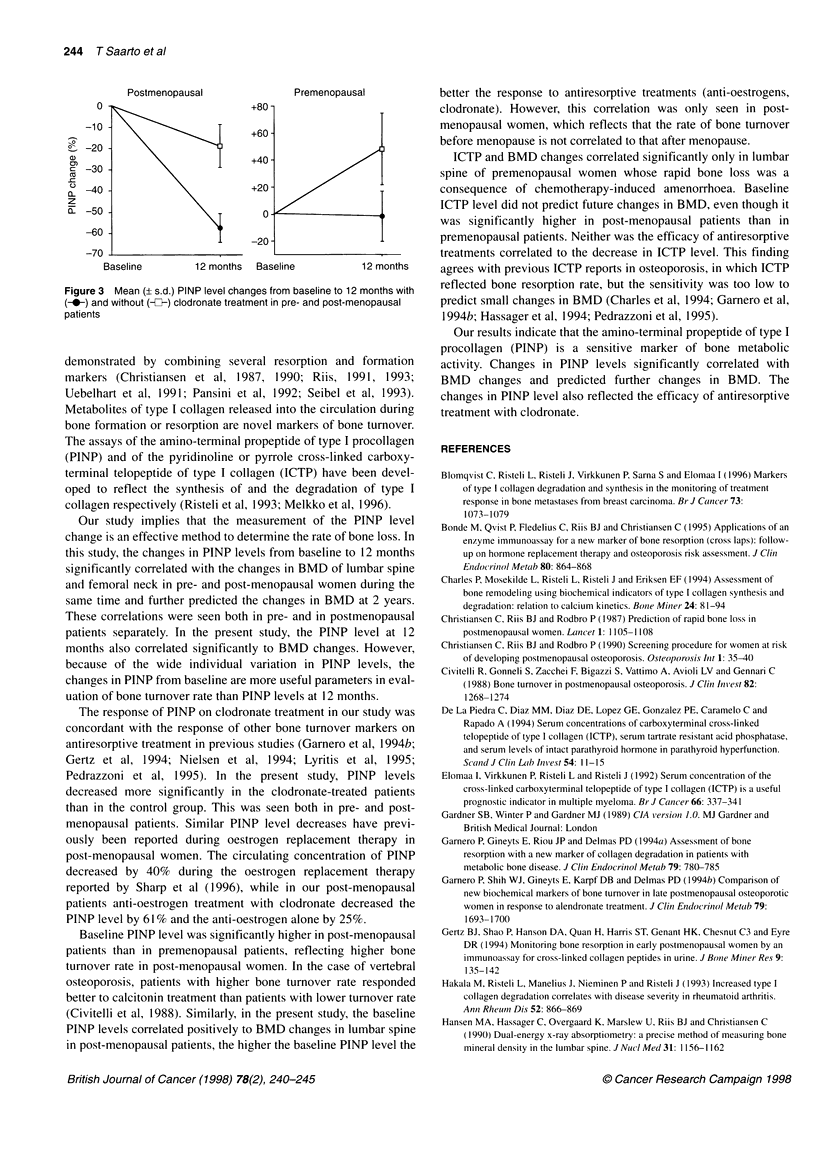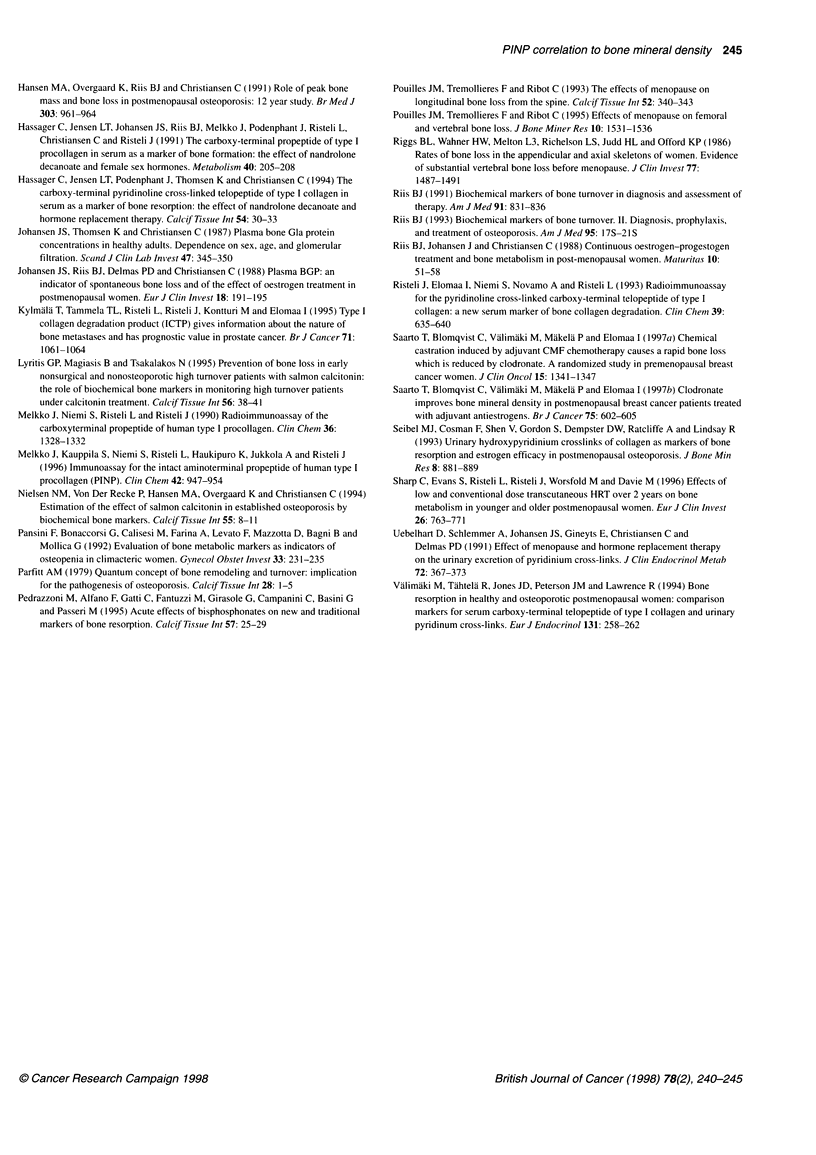# Aminoterminal propeptide of type I procollagen (PINP) correlates to bone loss and predicts the efficacy of antiresorptive therapy in pre- and post-menopausal non-metastatic breast cancer patients.

**DOI:** 10.1038/bjc.1998.471

**Published:** 1998-07

**Authors:** T. Saarto, C. Blomqvist, J. Risteli, L. Risteli, S. Sarna, I. Elomaa

**Affiliations:** Department of Oncology, University of Helsinki, Finland.

## Abstract

The aim of this study was to determine the correlation between changes in collagen metabolites (ICTP, mature cross-linked carboxy-terminal telopeptide of type I collagen; PINP, the amino-terminal propeptide of type I procollagen) and bone mineral density (BMD) in 206 pre- and post-menopausal breast cancer patients with non-metastatic disease. All patients received adjuvant cancer treatment--premenopausal patients chemotherapy and post-menopausal patients anti-oestrogens. In addition, the patients were also randomized to receive oral clodronate 1600 mg daily for 3 years. BMD was measured at baseline and at 1 and 2 years, the collagen metabolites at baseline and at 1 year. There was a highly significant negative correlation between the changes in PINP and BMD in lumbar spine and femoral neck from baseline to 12 months in all patients (r(s) = -0.68, P < 0.0001, and -0.45, P < 0.0001, respectively), and in pre- and post-menopausal patients separately. The changes in PINP levels at 12 months predict further changes in BMD at 24 months (r = -0.70, P < 0.0001, and -0.51, P < 0.0001, respectively). ICTP and BMD changes correlated significantly only in lumbar spine of premenopausal patients who developed rapid bone loss due to chemotherapy-induced amenorrhoea (r(s) = -0.34, P = 0.0003). The PINP levels at 12 months were significantly lower in the clodronate group than in the control group (P < 0.0001). Our results indicate that PINP is a sensitive marker of bone turnover rate. Changes in PINP levels significantly predicted changes in BMD and correlated with the antiresorptive efficacy of clodronate treatment.


British Journal of Cancer (1998) 78(2), 240–245
© 1998 Cancer Research Campaign

### Aminoterminal propeptide of type I procollagen (PINP) correlates to bone loss and predicts the efficacy of antiresorptive therapy in pre- and post-menopausal non-metastatic breast cancer patients

**T Saarto[1], C Blomqvist[1], J Risteli[2], L Risteli[2], S Sarna[3] and I Elomaa[1]**

[1]Department of Oncology, University of Helsinki; [2]Department of Medical Biochemistry, University of Oulu; [3]Department of Public Health, University of Helsinki, Helsinki, Finland

**Summary** The aim of this study was to determine the correlation between changes in collagen metabolites (ICTP, mature cross-linked carboxy-terminal telopeptide of type I collagen; PINP, the amino-terminal propeptide of type I procollagen) and bone mineral density (BMD) in 206 pre- and post-menopausal breast cancer patients with non-metastatic disease. All patients received adjuvant cancer treatment – premenopausal patients chemotherapy and post-menopausal patients anti-oestrogens. In addition, the patients were also randomized to receive oral clodronate 1600 mg daily for 3 years. BMD was measured at baseline and at 1 and 2 years, the collagen metabolites at baseline and at 1 year. There was a highly significant negative correlation between the changes in PINP and BMD in lumbar spine and femoral neck from baseline to 12 months in all patients ($r_s = -0.68$, $P < 0.0001$, and $-0.45$, $P < 0.0001$, respectively), and in pre- and post-menopausal patients separately. The changes in PINP levels at 12 months predict further changes in BMD at 24 months ($r_s = -0.70$, $P < 0.0001$, and $-0.51$, $P < 0.0001$, respectively). ICTP and BMD changes correlated significantly only in lumbar spine of premenopausal patients who developed rapid bone loss due to chemotherapy-induced amenorrhoea ($r_s = -0.34$, $P = 0.0003$). The PINP levels at 12 months were significantly lower in the clodronate group than in the control group ($P < 0.0001$). Our results indicate that PINP is a sensitive marker of bone turnover rate. Changes in PINP levels significantly predicted changes in BMD and correlated with the antiresorptive efficacy of clodronate treatment.

**Keywords:** adjuvant chemotherapy; breast neoplasm; collagen metabolites; post-menopausal osteoporosis

Post-menopausal osteoporosis is a common disorder. After menopause, bone turnover rate increases rapidly as a result of oestrogen deficiency. There is an imbalance between resorption and formation, the resorption exceeding the formation, with accelerated bone loss as a result (Parfitt, 1979). Early menopause, low bone mass at menopause and fast rate of bone loss after menopause are the risk factors of osteoporosis. BMD is the most accurate way of measuring bone mass and diagnosing osteoporosis. However, the rate of bone loss after menopause varies significantly from one woman to another and can not be predicted by a single BMD measurement (Christiansen et al, 1987, 1990; Hansen et al, 1991). Serial BMD measurements are needed, but because of the relatively small changes in bone mass per year in comparison with the precision of the measurement methods, it may take a long time to predict the rate of bone loss with BMD measurements (Riggs et al, 1986; Hansen et al, 1990; Pouilles et al, 1993, 1995).

An intriguing possibility would therefore be to use biochemical markers of bone turnover as indicators of the rate of bone loss. Both resorption (urinary excretions of hydroxyproline, pyridinoline cross-links of collagen, and cross-linked telopeptides of type I collagen) and formation markers of bone (circulating concentrations of alkaline phosphatase, osteocalcin and carboxy-terminal propeptide of type I procollagen) increase significantly after menopause. During hormone replacement therapy, these markers decrease to the premenopausal level (Johansen et al, 1987, 1988; Riis et al, 1988; Hassager et al, 1991; Riis, 1991, 1993; Uebelhart et al, 1991; Garnero et al, 1994*a*; Bonde et al, 1995). A decrease in bone turnover markers has also been documented during calcitonin and bisphosphonate treatment in post-menopausal osteoporosis (Garnero et al, 1994*b*; Gertz et al, 1994; Nielsen et al, 1994; Lyritis et al, 1995; Pedrazzoni et al, 1995). The best correlation between bone markers and bone loss rate, so far, has been demonstrated by combining measurements of several resorption and formation markers (Christiansen et al, 1987, 1990; Riis, 1991, 1993; Uebelhart et al, 1991; Pansini et al, 1992; Seibel et al, 1993).

As type I collagen is the most common protein in the skeleton, comprising about 90% of the organic matrix in bone tissue (Melkko et al, 1990), assays of the turnover of this protein could be good markers of bone turnover. A radioimmunoassay of the breakdown of mature type I collagen ICTP has recently been shown to be a sensitive marker of bone resorption in different diseases involving increased pathological degradation of collagen (Elomaa et al, 1992; Hakala et al, 1993; Risteli et al, 1993; De la Piedra et al, 1994; Kylmälä et al, 1995; Blomqvist et al, 1996). The results of ICTP as a resorption marker in post-menopausal osteoporosis have been conflicting (Charles et al, 1994; Garnero et al, 1994*b*; Hassager et al, 1994; Välimäki et al, 1994; Pedrazzoni et al, 1995). Amino-terminal propeptide of type I procollagen, PINP,

*Received 30 September 1996*
*Accepted 17 January 1997*

*Correspondence to:* I Elomaa, Department of Oncology, University of Helsinki, Haartmaninkatu 4, FIN-00290 Helsinki, Finland is a new marker intended to reflect the synthesis of type I collagen (Melkko et al, 1996). In osteoporosis, the changes in PINP levels follow the concentrations of osteocalcin and alkaline phosphatase, but differ from the values of PICP, which is the carboxy-terminal analogue of PINP (Sharp et al, 1996).

We have previously reported the results of BMD measurements in 206 primary early-stage breast cancer patients without metastases (Saarto et al, 1997*a* and *b*). Briefly, bone loss in premenopausal patients correlated significantly with the ovarian dysfunction induced by adjuvant chemotherapy. The most marked bone loss was seen in those patients rendered amenorrhoeic by chemotherapy. Clodronate significantly reduced the bone loss both in lumbar spine and in femoral neck in all premenopausal women (in the control group – 5.9% and – 0.2% and in the clodronate group – 2.2% and + 0.9% respectively) and in amenorrhoeic patients separately (in the control group – 9.5% and – 4.6% and in the clodronate group – 5.9% and – 0.4% respectively). In post-menopausal patients, anti-oestrogen treatment with tamoxifen or toremifene seemed to prevent the development of post-menopausal osteoporosis. Clodronate treatment significantly improved BMD of lumbar spine and femoral neck in post-menopausal patients (in the control group – 0.5% + 0.5% and in the clodronate group + 2.9% and + 3.7% respectively). Here, we wanted to find out in the same patients whether changes in PINP and ICTP levels may (1) correlate with BMD behaviour and (2) predict the efficacy of clodronate therapy.

### MATERIAL AND METHODS

### Patients and methods

The study population consisted of 206 pre- and post-menopausal women with operable breast cancer and histologically proven axillary metastases, without haematogenic metastases. Eligible for the analyses were patients who were disease free at the time of measurement of BMD and collagen metabolites. In addition, patients having bone metastases within 6 months after measurement of BMD and collagen metabolites were excluded from the analyses. Baseline adjuvant cancer treatment was six cycles of CMF chemotherapy for premenopausal patients (cyclophosphamide 600 mg $m^{-2}$, methotrexate 40 mg $m^{-2}$ and 5-fluorouracil 600 mg $m^{-2}$) and anti-oestrogens for post-menopausal patients (tamoxifen 20 mg or toremifene 60 mg per day) for 3 years. All patients underwent surgery with axillary evacuation and total mastectomy or breast-conserving resection and post-operative radiotherapy. All patients were randomized to receive oral clodronate (Bonefos, Leiras) 1600 mg daily for 3 years or to a control group. After chemotherapy, the premenopausal patients were divided into two groups according to menstrual function at 1 year of follow-up: menstruating (regularly or irregularly) or amenorrhoeic patients. Fast bone losers were defined as patients who lost BMD by more than 3% per year. The BMD changes during the trial of 2 years have been previously reported (Saarto et al, 1997*a* and *b*).

Biochemical measurements were performed at the start of the study and at 12 months. All serum samples were stored at – 20°C. ICTP and PINP reflect degradation and synthesis, respectively, of type I collagen, the predominant collagen in bone matrix. The methods for the PINP and ICTP assays have been described elsewhere (Risteli et al, 1993; Melkko et al, 1996). The reference interval of ICTP for adult women is 1.7–4.6 μg $l^{-1}$, and that of PINP 19–84 μg $l^{-1}$. The intra- and interassay coefficients of variation are 3.1–8.5% for PINP, and 2.8–6.2% and 4.1–7.9% for ICTP respectively (Risteli et al, 1993; Melkko et al, 1996). Bone mineral density (BMD, g $cm^{-2}$) was measured by dual-energy X-ray absorptiometry (DXA) using a Hologic QDR-1000 densitometer (Hologic, Waltham, MA, USA). BMD was measured at the lumbar vertebrae (L1–L4) and femoral neck in the right femoral area before initiation of therapy and at 1 and 2 years. The coefficients of variation for precision of the BMD measurements in the lumbar vertebrae and femoral neck were 0.9% and 1.2% respectively.

**Table 1** Correlation between baseline levels of collagen markers and baseline level of BMD or changes in BMD at 1 and 2 years. Spearman's rank-order correlation and 95% confidence intervals

| Baseline collagen markers | Baseline BMD | | BMD change in LS | | BMD change in FN | |
|---|---|---|---|---|---|---|
| | LS | FN | 1 year | 2 year | 1 year | 2 year |
| *All patients* | | | | | | |
| PINP | $r_s = -0.26$ (– 0.39, – 0.12) ($P = 0.0002$) | $r_s = -0.23$ (– 0.36, – 0.09) ($P = 0.0009$) | $r_s = 0.32$ (0.19, 0.44) ($P < 0.0001$) | $r_s = 0.30$ (0.16, 0.43) ($P < 0.0001$) | NS | NS |
| ICTP | NS | NS | $r_s = 0.18$ (0.41, 0.31) ($P = 0.01$) | NS | NS | NS |
| *Premenopausal patients* | | | | | | |
| PINP | NS | NS | NS | NS | NS | NS |
| ICTP | NS | NS | NS | NS | NS | NS |
| *Post-menopausal patients* | | | | | | |
| PINP | $r_s = -0.27$ (– 0.45, – 0.07) ($P = 0.01$) | NS | $r_s = 0.32$ (0.12, 0.50) ($P = 0.002$) | $r_s = 0.34$ (0.13, 0.52) ($P = 0.002$) | NS | NS |
| ICTP | NS | NS | NS | NS | NS | NS |

LS, lumbar spine; FN, femoral neck.

© Cancer Research Campaign 1998

Table 2 Correlation between BMD changes at 1 and 2 years and changes in the levels of collagen markers from baseline to those at 12 months. Spearman's rank-order correlation and 95% confidence intervals

| Change in collagen markers | BMD change in LS | | BMD change in FN | |
|---|---|---|---|---|
| | 1 year | 2 year | 1 year | 2 year |
| *All patients* | | | | |
| PINP | $r_s = -0.68$ (− 0.75, − 0.60) ($P < 0.0001$) | $r_s = -0.70$ (− 0.77, − 0.61) ($P < 0.0001$) | $r_s = -0.45$ (− 0.56, − 0.33) ($P < 0.0001$) | $r_s = -0.51$ (− 0.61, − 0.39) ($P < 0.0001$) |
| ICTP | $r_s = -0.27$ (− 0.40, − 0.13) ($P = 0.0001$) | $r_s = -0.31$ (− 0.43, − 0.16) ($P < 0.0001$) | NS | $r_s = -0.25$ (− 0.39, − 0.10) ($P = 0.0009$) |
| *Premenopausal* | | | | |
| PINP | $r_s = -0.60$ (− 0.71, − 0.46) ($P < 0.0001$) | $r_s = -0.60$ (− 0.72, − 0.45) ($P < 0.0001$) | $r_s = -0.38$ (− 0.53, − 0.21) ($P < 0.0001$) | $r_s = -0.43$ − 0.58, − 0.23) ($P < 0.0001$) |
| ICTP | $r_s = -0.34$ (− 0.50, − 0.16) ($P = 0.0003$) | $r_s = -0.38$ (− 0.54, − 0.19) ($P = 0.0002$) | NS | $r_s = -0.30$ (− 0.48, − 0.10) ($P = 0.003$) |
| *Post-menopausal* | | | | |
| PINP | $r_s = -0.59$ (− 0.71, − 0.43) ($P < 0.0001$) | $r_s = -0.62$ (− 0.74, − 0.46) ($P < 0.0001$) | $r_s = -0.34$ (− 0.51, − 0.14) ($P = 0.001$) | $r_s = -0.52$ (− 0.67, − 0.34) ($P < 0.0001$) |
| ICTP | NS | NS | NS | NS |

LS, lumbar spine; FN, femoral neck.

### Statistical methods

Of the 206 eligible patients, nine patients had missing values for baseline PINP level and two at 12 months, ten patients had missing values for baseline ICTP and three at 12 months. BMD measurement was available in 179 patients at 2 years after the start of the study. Cases with missing laboratory values were excluded from those analyses only when these values were needed. Analyses were performed for all patients and for the pre- and post-menopausal patients separately. The differences in marker values between premenopausal patients with preserved menstruation or with induced amenorrhoea and the effect of clodronate treatment on marker change were tested with the Mann–Whitney test. The correlations between marker levels and the BMD were assessed using Spearman's rank-order correlation coefficient ($r_s$). Marker change was calculated as marker level at 12 months of follow-up divided by the baseline level. Confidence intervals (95%) for $r_s$ were calculated with the CIA statistical software (Gardner et al, 1989). Because of the problems of multiple comparisons, the significance level was set at 0.01.

### RESULTS

### Correlation between type I collagen metabolites and BMD at baseline

The median PINP value at baseline was 42.8 μg $l^{-1}$ (range 11.4–169.4 μg $l^{-1}$), in premenopausal patients 40.6 μg $l^{-1}$ (range 11.4–90.6 μg $l^{-1}$) and in post-menopausal patients 45.4 μg $l^{-1}$ (range 21.2–169.4 μg $l^{-1}$). Baseline PINP level was significantly higher in the post-menopausal than in the premenopausal patients ($P = 0.008$), but there was no baseline differences between the premenopausal patients who preserved menstruation and those who became amenorrhoeic after chemotherapy. Baseline PINP values were within the reference interval in 191 patients (97%). The baseline PINP concentration correlated negatively to baseline BMD in lumbar spine and in femoral neck ($r_s = -0.25$, $P = 0.0002$, and $r_s = -0.23$, $P = 0.0009$, respectively). The negative correlation between baseline lumbar spine BMD and PINP level was also significant in post-menopausal patients, but not in premenopausal patients (Table 1).

The median concentration of ICTP at baseline was 3.8 μg $l^{-1}$ (range 1.6–17.2 μg $l^{-1}$), in premenopausal patients 3.5 μg $l^{-1}$ (range 1.6–8.3 μg $l^{-1}$) and in post-menopausal 4.0 μg $l^{-1}$ (range 1.8–17.2 μg $l^{-1}$). Baseline ICTP level was significantly higher in post-menopausal than in premenopausal patients ($P = 0.004$), with no baseline differences between the premenopausal patients who, after chemotherapy, menstruated or were amenorrhoeic. The baseline ICTP value was within the reference interval in 153 patients (78%). The baseline ICTP concentrations did not correlate with baseline BMD (Table 1).

### Prediction of bone loss by type I collagen metabolites at baseline

The baseline PINP level correlated positively with the BMD changes in the lumbar spine at 1 and 2 years ($r_s = 0.32$, $P < 0.0001$, and $r_s = 0.30$, $P < 0.0001$, respectively), but not with those in the femoral neck. The significant correlations with BMD in lumbar spine at 1 and 2 years were also seen in the group of post-menopausal, but not in that of premenopausal, patients. A marginal positive correlation was also seen between the baseline ICTP level and the BMD change in lumbar spine at 1 year ($r_s = 0.18$, $P = 0.01$) (Table 1).

© Cancer Research Campaign 1998

**Figure 1** Correlation between changes in lumbar spine BMD and PINP levels from baseline to 12 months in all patients (mean ± s.d.). Regression lines in all patients $y = 117\,842 - 9886^*x$, $r^2 = 0.414$; in premenopausal patients $y = 116\,337 - 9329^*x$, $r^2 = 0.289$; in post-menopausal patients $y = 116\,119 - 8709^*x$, $r^2 = 0.360$; in clodronate-treated patients $y = 112\,583 - 6893^*x$, $r^2 = 0.320$; and in control patients $y = 123\,586 - 12\,765^*x$, $r^2 = 0.436$

**Figure 2** Correlation between PINP level changes and changes in lumbar spine BMD from baseline to 12 months in amenorrhoeic and menstruating premenopausal patients (mean ± s.d.)

### Correlation between type I collagen metabolites at 12 months and BMD changes

PINP levels at 12 months correlated significantly negatively to BMD changes at 1 and 2 years in lumbar spine ($r_s = -0.54$, $P < 0.0001$, and $r_s = -0.59$, $P < 0.0001$) and femoral neck ($r_s = -0.39$, $P < 0.0001$, and $r_s = -0.47$, $P < 0.0001$) in all patients; in the premenopausal women in lumbar spine ($r_s = -0.46$, $P < 0.0001$, and $r_s = -0.50$, $P < 0.0001$) and femoral neck ($r_s = -0.37$, $P < 0.0001$, and $r_s = -0.39$, $P < 0.0001$); and in post-menopausal patients in lumbar spine ($r_s = -0.45$, $P < 0.0001$, and $r_s = -0.51$, $P < 0.0001$) and femoral neck ($r_s = -0.23$, $P = 0.026$, and $r_s = -0.48$, $P < 0.0001$).

A marginal negative correlation was also seen in premenopausal patients between ICTP levels at 12 months and BMD changes at 1 and 2 years in lumbar spine ($r_s = -0.26$, $P = 0.005$, and $-0.34$, $P = 0.0007$) and in femoral neck ($r_s = -0.20$, $P = 0.03$ and $-0.29$, $P = 0.004$), but not in post-menopausal patients.

### Prediction of bone loss by changes in type I collagen metabolites

The Spearman's rank-order correlation coefficients between the changes observed in the markers from baseline to 12 months and the BMD changes in lumbar spine and femoral neck at 1 and 2 years are shown in Table 2 and Figure 1. There was a highly significant negative correlation between PINP changes from baseline to 12 months and BMD changes to 1 and 2 years in all patients in lumbar spine ($r_s = -0.68$, $P < 0.0001$, and $-0.70$, $P < 0.0001$) and femoral neck ($r_s = -0.45$, $P < 0.0001$, and $-0.51$, $P < 0.0001$), and in pre- and post-menopausal patients separately. The median change in PINP level in all patients was − 6.7 μg $l^{-1}$ (− 18.3%), in premenopausal patients + 0.6 μg $l^{-1}$ (+ 1.4%), in post-menopausal patients − 17.6 μg $l^{-1}$ (− 41.8%) and separately in menstruating and amenorrhoeic premenopausal patients − 4.6 μg $l^{-1}$ (− 15.7%) and + 28.4 μg $l^{-1}$ (+ 61.0%) respectively (Figure 2).

ICTP change correlated significantly negatively with lumbar spine BMD change at 1 and 2 years in all patients ($r_s = -0.27$, $P = 0.0001$, and $-0.31$, $P < 0.0001$) and in premenopausal patients ($r_s = -0.34$, $P = 0.0003$, and $-0.38$, $P = 0.0002$), but with femoral neck only at 2 years. No correlation was seen in post-menopausal patients (Table 2). The median change in ICTP levels in all patients was − 0.7 μg $l^{-1}$ (− 22.8%), in premenopausal patients − 0.3 μg $l^{-1}$ (− 14.8%), in post-menopausal patients − 1.0 μg $l^{-1}$ (− 25.0%) and in menstruating and amenorrhoeic premenopausal patients − 0.6 μg $l^{-1}$ (− 20.0%) and + 0.05 μg $l^{-1}$ (+ 1.5%) respectively.

### Clodronate vs control group

Baseline values of PINP were similar in the clodronate and control groups. PINP levels at 12 months were significantly lower in the clodronate group than in the control group, in all patients and also in pre- and post-menopausal patients ($P < 0.0001$, 0.0003 and < 0.0001 respectively). In all patients, median PINP levels decreased in the clodronate and control groups by 14.6 μg $l^{-1}$ (− 38.7%) and 0.7 μg $l^{-1}$ (− 1.7%) respectively ($P < 0.0001$). In premenopausal patients, median PINP levels decreased with clodronate by 5.6 μg $l^{-1}$ (− 16.8%), but increased without it by 9.2 μg $l^{-1}$ (+ 29.5%) ($P = 0.0005$): in menstruating patients, the corresponding changes were − 11.6 μg $l^{-1}$ (− 27.6%) with and − 2.1 μg $l^{-1}$ (− 3.6%) without clodronate, and in amenorrheic patients + 0.7 μg $l^{-1}$ (+ 1.8%) with and + 34.5 μg $l^{-1}$ (+ 83.3%) without clodronate ($P = 0.003$ and 0.007). In post-menopausal patients, median PINP levels decreased by 30.8 μg $l^{-1}$ (− 60.8%) with and by 12.4 μg $l^{-1}$ (− 25.2%) without clodronate ($P < 0.0001$) (Figure 3).

The clodronate and control groups did not differ from each other by baseline or 12-month ICTP levels, nor by changes in ICTP levels from the baseline to 12 months.

### DISCUSSION

In the development of osteoporosis, the rate of bone loss after menopause is at least as important as bone mass at menopause. Women who have had rapid bone loss at menopause lose, on average, twice the amount of bone mass over 12 years after menopause than normal bone losers (Hansen et al, 1991). Several attempts have been made to identify rapid bone losers by measuring biochemical markers of bone turnover. The best correlation between bone markers and bone loss rate, so far, has been

© Cancer Research Campaign 1998

**Figure 3** Mean (± s.d.) PINP level changes from baseline to 12 months with (-●-) and without (-□-) clodronate treatment in pre- and post-menopausal patients demonstrated by combining several resorption and formation markers (Christiansen et al, 1987, 1990; Riis, 1991, 1993; Uebelhart et al, 1991; Pansini et al, 1992; Seibel et al, 1993). Metabolites of type I collagen released into the circulation during bone formation or resorption are novel markers of bone turnover. The assays of the amino-terminal propeptide of type I procollagen (PINP) and of the pyridinoline or pyrrole cross-linked carboxy-terminal telopeptide of type I collagen (ICTP) have been developed to reflect the synthesis of and the degradation of type I collagen respectively (Risteli et al, 1993; Melkko et al, 1996).

Our study implies that the measurement of the PINP level change is an effective method to determine the rate of bone loss. In this study, the changes in PINP levels from baseline to 12 months significantly correlated with the changes in BMD of lumbar spine and femoral neck in pre- and post-menopausal women during the same time and further predicted the changes in BMD at 2 years. These correlations were seen both in pre- and in postmenopausal patients separately. In the present study, the PINP level at 12 months also correlated significantly to BMD changes. However, because of the wide individual variation in PINP levels, the changes in PINP from baseline are more useful parameters in evaluation of bone turnover rate than PINP levels at 12 months.

The response of PINP on clodronate treatment in our study was concordant with the response of other bone turnover markers on antiresorptive treatment in previous studies (Garnero et al, 1994*b*; Gertz et al, 1994; Nielsen et al, 1994; Lyritis et al, 1995; Pedrazzoni et al, 1995). In the present study, PINP levels decreased more significantly in the clodronate-treated patients than in the control group. This was seen both in pre- and post-menopausal patients. Similar PINP level decreases have previously been reported during oestrogen replacement therapy in post-menopausal women. The circulating concentration of PINP decreased by 40% during the oestrogen replacement therapy reported by Sharp et al (1996), while in our post-menopausal patients anti-oestrogen treatment with clodronate decreased the PINP level by 61% and the anti-oestrogen alone by 25%.

Baseline PINP level was significantly higher in post-menopausal patients than in premenopausal patients, reflecting higher bone turnover rate in post-menopausal women. In the case of vertebral osteoporosis, patients with higher bone turnover rate responded better to calcitonin treatment than patients with lower turnover rate (Civitelli et al, 1988). Similarly, in the present study, the baseline PINP levels correlated positively to BMD changes in lumbar spine in post-menopausal patients, the higher the baseline PINP level the better the response to antiresorptive treatments (anti-oestrogens, clodronate). However, this correlation was only seen in post-menopausal women, which reflects that the rate of bone turnover before menopause is not correlated to that after menopause.

ICTP and BMD changes correlated significantly only in lumbar spine of premenopausal women whose rapid bone loss was a consequence of chemotherapy-induced amenorrhoea. Baseline ICTP level did not predict future changes in BMD, even though it was significantly higher in post-menopausal patients than in premenopausal patients. Neither was the efficacy of antiresorptive treatments correlated to the decrease in ICTP level. This finding agrees with previous ICTP reports in osteoporosis, in which ICTP reflected bone resorption rate, but the sensitivity was too low to predict small changes in BMD (Charles et al, 1994; Garnero et al, 1994*b*; Hassager et al, 1994; Pedrazzoni et al, 1995).

Our results indicate that the amino-terminal propeptide of type I procollagen (PINP) is a sensitive marker of bone metabolic activity. Changes in PINP levels significantly correlated with BMD changes and predicted further changes in BMD. The changes in PINP level also reflected the efficacy of antiresorptive treatment with clodronate.

### REFERENCES

Blomqvist C, Risteli L, Risteli J, Virkkunen P, Sarna S and Elomaa I (1996) Markers of type I collagen degradation and synthesis in the monitoring of treatment response in bone metastases from breast carcinoma. *Br J Cancer* **73**: 1073–1079

Bonde M, Qvist P, Fledelius C, Riis BJ and Christiansen C (1995) Applications of an enzyme immunoassay for a new marker of bone resorption (cross laps): follow-up on hormone replacement therapy and osteoporosis risk assessment. *J Clin Endocrinol Metab* **80**: 864–868

Charles P, Mosekilde L, Risteli L, Risteli J and Eriksen EF (1994) Assessment of bone remodeling using biochemical indicators of type I collagen synthesis and degradation: relation to calcium kinetics. *Bone Miner* **24**: 81–94

Christiansen C, Riis BJ and Rodbro P (1987) Prediction of rapid bone loss in postmenopausal women. *Lancet* **1**: 1105–1108

Christiansen C, Riis BJ and Rodbro P (1990) Screening procedure for women at risk of developing postmenopausal osteoporosis. *Osteoporosis Int* **1**: 35–40

Civitelli R, Gonneli S, Zacchei F, Bigazzi S, Vattimo A, Avioli LV and Gennari C (1988) Bone turnover in postmenopausal osteoporosis. *J Clin Invest* **82**: 1268–1274

De La Piedra C, Diaz MM, Diaz DE, Lopez GE, Gonzalez PE, Caramelo C and Rapado A (1994) Serum concentrations of carboxyterminal cross-linked telopeptide of type I collagen (ICTP), serum tartrate resistant acid phosphatase, and serum levels of intact parathyroid hormone in parathyroid hyperfunction. *Scand J Clin Lab Invest* **54**: 11–15

Elomaa I, Virkkunen P, Risteli L and Risteli J (1992) Serum concentration of the cross-linked carboxyterminal telopeptide of type I collagen (ICTP) is a useful prognostic indicator in multiple myeloma. *Br J Cancer* **66**: 337–341

Gardner SB, Winter P and Gardner MJ (1989) *CIA version 1.0.* MJ Gardner and British Medical Journal: London

Garnero P, Gineyts E, Riou JP and Delmas PD (1994*a*) Assessment of bone resorption with a new marker of collagen degradation in patients with metabolic bone disease. *J Clin Endocrinol Metab* **79**: 780–785

Garnero P, Shih WJ, Gineyts E, Karpf DB and Delmas PD (1994*b*) Comparison of new biochemical markers of bone turnover in late postmenopausal osteoporotic women in response to alendronate treatment. *J Clin Endocrinol Metab* **79**: 1693–1700

Gertz BJ, Shao P, Hanson DA, Quan H, Harris ST, Genant HK, Chesnut C3 and Eyre DR (1994) Monitoring bone resorption in early postmenopausal women by an immunoassay for cross-linked collagen peptides in urine. *J Bone Miner Res* **9**: 135–142

Hakala M, Risteli L, Manelius J, Nieminen P and Risteli J (1993) Increased type I collagen degradation correlates with disease severity in rheumatoid arthritis. *Ann Rheum Dis* **52**: 866–869

Hansen MA, Hassager C, Overgaard K, Marslew U, Riis BJ and Christiansen C (1990) Dual-energy x-ray absorptiometry: a precise method of measuring bone mineral density in the lumbar spine. *J Nucl Med* **31**: 1156–1162

© Cancer Research Campaign 1998

Hansen MA, Overgaard K, Riis BJ and Christiansen C (1991) Role of peak bone mass and bone loss in postmenopausal osteoporosis: 12 year study. *Br Med J* **303**: 961–964

Hassager C, Jensen LT, Johansen JS, Riis BJ, Melkko J, Podenphant J, Risteli L, Christiansen C and Risteli J (1991) The carboxy-terminal propeptide of type I procollagen in serum as a marker of bone formation: the effect of nandrolone decanoate and female sex hormones. *Metabolism* **40**: 205–208

Hassager C, Jensen LT, Podenphant J, Thomsen K and Christiansen C (1994) The carboxy-terminal pyridinoline cross-linked telopeptide of type I collagen in serum as a marker of bone resorption: the effect of nandrolone decanoate and hormone replacement therapy. *Calcif Tissue Int* **54**: 30–33

Johansen JS, Thomsen K and Christiansen C (1987) Plasma bone Gla protein concentrations in healthy adults. Dependence on sex, age, and glomerular filtration. *Scand J Clin Lab Invest* **47**: 345–350

Johansen JS, Riis BJ, Delmas PD and Christiansen C (1988) Plasma BGP: an indicator of spontaneous bone loss and of the effect of oestrogen treatment in postmenopausal women. *Eur J Clin Invest* **18**: 191–195

Kylmälä T, Tammela TL, Risteli L, Risteli J, Kontturi M and Elomaa I (1995) Type I collagen degradation product (ICTP) gives information about the nature of bone metastases and has prognostic value in prostate cancer. *Br J Cancer* **71**: 1061–1064

Lyritis GP, Magiasis B and Tsakalakos N (1995) Prevention of bone loss in early nonsurgical and nonosteoporotic high turnover patients with salmon calcitonin: the role of biochemical bone markers in monitoring high turnover patients under calcitonin treatment. *Calcif Tissue Int* **56**: 38–41

Melkko J, Niemi S, Risteli L and Risteli J (1990) Radioimmunoassay of the carboxyterminal propeptide of human type I procollagen. *Clin Chem* **36**: 1328–1332

Melkko J, Kauppila S, Niemi S, Risteli L, Haukipuro K, Jukkola A and Risteli J (1996) Immunoassay for the intact aminoterminal propeptide of human type I procollagen (PINP). *Clin Chem* **42**: 947–954

Nielsen NM, Von Der Recke P, Hansen MA, Overgaard K and Christiansen C (1994) Estimation of the effect of salmon calcitonin in established osteoporosis by biochemical bone markers. *Calcif Tissue Int* **55**: 8–11

Pansini F, Bonaccorsi G, Calisesi M, Farina A, Levato F, Mazzotta D, Bagni B and Mollica G (1992) Evaluation of bone metabolic markers as indicators of osteopenia in climacteric women. *Gynecol Obstet Invest* **33**: 231–235

Parfitt AM (1979) Quantum concept of bone remodeling and turnover: implication for the pathogenesis of osteoporosis. *Calcif Tissue Int* **28**: 1–5

Pedrazzoni M, Alfano F, Gatti C, Fantuzzi M, Girasole G, Campanini C, Basini G and Passeri M (1995) Acute effects of bisphosphonates on new and traditional markers of bone resorption. *Calcif Tissue Int* **57**: 25–29

Pouilles JM, Tremollieres F and Ribot C (1993) The effects of menopause on longitudinal bone loss from the spine. *Calcif Tissue Int* **52**: 340–343

Pouilles JM, Tremollieres F and Ribot C (1995) Effects of menopause on femoral and vertebral bone loss. *J Bone Miner Res* **10**: 1531–1536

Riggs BL, Wahner HW, Melton L3, Richelson LS, Judd HL and Offord KP (1986) Rates of bone loss in the appendicular and axial skeletons of women. Evidence of substantial vertebral bone loss before menopause. *J Clin Invest* **77**: 1487–1491

Riis BJ (1991) Biochemical markers of bone turnover in diagnosis and assessment of therapy. *Am J Med* **91**: 831–836

Riis BJ (1993) Biochemical markers of bone turnover. II. Diagnosis, prophylaxis, and treatment of osteoporosis. *Am J Med* **95**: 17S–21S

Riis BJ, Johansen J and Christiansen C (1988) Continuous oestrogen–progestogen treatment and bone metabolism in post-menopausal women. *Maturitas* **10**: 51–58

Risteli J, Elomaa I, Niemi S, Novamo A and Risteli L (1993) Radioimmunoassay for the pyridinoline cross-linked carboxy-terminal telopeptide of type I collagen: a new serum marker of bone collagen degradation. *Clin Chem* **39**: 635–640

Saarto T, Blomqvist C, Välimäki M, Mäkelä P and Elomaa I (1997*a*) Chemical castration induced by adjuvant CMF chemotherapy causes a rapid bone loss which is reduced by clodronate. A randomized study in premenopausal breast cancer women. *J Clin Oncol* **15**: 1341–1347

Saarto T, Blomqvist C, Välimäki M, Mäkelä P and Elomaa I (1997*b*) Clodronate improves bone mineral density in postmenopausal breast cancer patients treated with adjuvant antiestrogens. *Br J Cancer* **75**: 602–605

Seibel MJ, Cosman F, Shen V, Gordon S, Dempster DW, Ratcliffe A and Lindsay R (1993) Urinary hydroxypyridinium crosslinks of collagen as markers of bone resorption and estrogen efficacy in postmenopausal osteoporosis. *J Bone Min Res* **8**: 881–889

Sharp C, Evans S, Risteli L, Risteli J, Worsfold M and Davie M (1996) Effects of low and conventional dose transcutaneous HRT over 2 years on bone metabolism in younger and older postmenopausal women. *Eur J Clin Invest* **26**: 763–771

Uebelhart D, Schlemmer A, Johansen JS, Gineyts E, Christiansen C and Delmas PD (1991) Effect of menopause and hormone replacement therapy on the urinary excretion of pyridinium cross-links. *J Clin Endocrinol Metab* **72**: 367–373

Välimäki M, Tähtelä R, Jones JD, Peterson JM and Lawrence R (1994) Bone resorption in healthy and osteoporotic postmenopausal women: comparison markers for serum carboxy-terminal telopeptide of type I collagen and urinary pyridinum cross-links. *Eur J Endocrinol* **131**: 258–262

© Cancer Research Campaign 1998